# α-Cellulose Fibers of Paper-Waste Origin Surface-Modified with Fe_3_O_4_ and Thiolated-Chitosan for Efficacious Immobilization of Laccase

**DOI:** 10.3390/polym13040581

**Published:** 2021-02-15

**Authors:** Gajanan S. Ghodake, Surendra K. Shinde, Ganesh D. Saratale, Rijuta G. Saratale, Min Kim, Seung-Cheol Jee, Dae-Young Kim, Jung-Suk Sung, Avinash A. Kadam

**Affiliations:** 1 Department of Biological and Environmental Science, Dongguk University-Seoul, Ilsandong-gu, Goyang-si, Gyonggido, Seoul 10326, Korea; ghodakegs@gmail.com (G.S.G.); surendrashinde.phy@gmail.com (S.K.S.); sbpkim@dongguk.edu (D.-Y.K.); 2 Department of Food Science and Biotechnology, Dongguk University-Seoul, Ilsandong-gu, Goyang-si, Gyeonggi-do, Seoul 10326, Korea; gdsaratale@gmail.com; 3 Research Institute of Biotechnology and Medical Converged Science, Dongguk University-Seoul, Ilsandong-gu, Goyang-si, Gyeonggi-do, Seoul 10326, Korea; rijutaganesh@gmail.com; 4 Department of Life Science, College of Life Science and Biotechnology, Dongguk University-Seoul, Ilsandong-gu, Goyang-si, Gyonggido, Seoul 10326, Korea; pipikimmin@naver.com (M.K.); markjee@naver.com (S.-C.J.); sungjs@dongguk.edu (J.-S.S.)

**Keywords:** α-Cellulose, waste-paper-biomass, chitosan, laccase immobilization, super-magnetic

## Abstract

The utilization of waste-paper-biomass for extraction of important α-cellulose biopolymer, and modification of extracted α-cellulose for application in enzyme immobilization can be extremely vital for green circular bio-economy. Thus, in this study, α-cellulose fibers were super-magnetized (Fe_3_O_4_), grafted with chitosan (CTNs), and thiol (-SH) modified for laccase immobilization. The developed material was characterized by high-resolution transmission electron microscopy (HR-TEM), HR-TEM energy dispersive X-ray spectroscopy (HR-TEM-EDS), X-ray diffraction (XRD), vibrating sample magnetometer (VSM), X-ray photoelectron spectroscopy (XPS), and Fourier transform infrared spectroscopy (FT-IR) analyses. Laccase immobilized on α-Cellulose-Fe_3_O_4_-CTNs (α-Cellulose-Fe_3_O_4_-CTNs-Laccase) gave significant activity recovery (99.16%) and laccase loading potential (169.36 mg/g). The α-Cellulose-Fe_3_O_4_-CTNs-Laccase displayed excellent stabilities for temperature, pH, and storage time. The α-Cellulose-Fe_3_O_4_-CTNs-Laccase applied in repeated cycles shown remarkable consistency of activity retention for 10 cycles. After the 10th cycle, α-Cellulose-Fe_3_O_4_-CTNs possessed 80.65% relative activity. Furthermore, α-Cellulose-Fe_3_O_4_-CTNs-Laccase shown excellent degradation of pharmaceutical contaminant sulfamethoxazole (SMX). The SMX degradation by α-Cellulose-Fe_3_O_4_-CTNs-Laccase was found optimum at incubation time (20 h), pH (3), temperatures (30 °C), and shaking conditions (200 rpm). Finally, α-Cellulose-Fe_3_O_4_-CTNs-Laccase gave repeated degradation of SMX. Thus, this study presents a novel, waste-derived, highly capable, and super-magnetic nanocomposite for enzyme immobilization applications.

## 1. Introduction

Paper and cardboards related waste count near about 30% of the total urban solid waste produced worldwide [1]. Despite recycling rates is higher in most of the developed countries, solid paper waste [2], and food waste [3,4], remained as a significant concern to the landfill sites. At the same time, the growing population worldwide, and the emergence of linear bio economies in addition to the growing demand for end-use products causing over-exploitation of natural resources at a rapid pace [5,6]. On average about 55% of the slurry from the paper industry globally are made from the secondary fibers called recycled fibers; however, for some other paper grades, such as cardboard or newspaper, this percentage can be close to about 99%, while achieving zero waste [7]. Therefore, sustainable practices to extract cellulosic fibers from waste wood, paper waste, and cardboard waste are suggested to compensate the principle called the “cellulose gap” include decrease dependence on wood cellulose and increase material reuse, and circular bio-economy practices [8,9,10].

Though cellulosic fibers share only a small stake in the manmade fiber sector, their unique properties related to moisture absorption [11], mechanical strength [12], biocompatibility, and some other functionalities renders cellulosic fibers are indispensable for various processes, products, and ultimately numerous technologies [13,14]. The consumption of cellulosic per capita is anticipated to be increasing constantly [1], but the cultivation of trees as the major source of cellulosic fibers have limited scope to expand [15,16,17]. Therefore, recycling cellulose from waste is generally considered as a more sustainable alternative from an environmental protection point of view [18], meanwhile, it allows the reuse of the cellulose fibers to produce new paper, cardboards, value-added materials (α-cellulose, cellulose nanofibers, nanocomposites) and, thus, minimize the consumption load on natural resources, and simultaneously reduces the generation of waste [19].

The term “circular economy” was predominantly developed by the Ellen MacArthur Foundation [20]. This can be archived by two major routes: Those from where biodegradable waste having nutrients are suitable to return to nature or reuse [21,22,23] and non-degradable waste needs cycling for biopolymers to reduce pollution, for the paper sector as well [24]. However, cellulose fiber processing from paper pulp involves different chemical–physical processes and specific operating conditions to facilitate the dissolution and regeneration of cellulose materials [25,26,27,28,29]. The extraction of cellulose from paper waste and its utilization has to be made evident to exert an excellent response on the circular economy projects worldwide with the involvement of sustainability [30]. Our group has recently reported a greener method for the production of α-cellulosic fibers from print paper waste and their utilization in the remediation of nanomaterial waste [31,32] and preparation of super-magnetic α-cellulose fiber-chitosan composite for covalent laccase immobilization [33].

An alternative method for enzyme immobilization using biocompatible nano-supports is an attractive approach for the improved stability and economic feasibility of potential biocatalysts [34,35,36]. The immobilization of laccase and exploring its environmental applications has received enormous attention due to ease of separation, long-term stability, and reuse of the immobilized biocatalysts [37,38]. Thiolation of the CTNs surfaces is an emerging methodology for enzymes, peptides, and proteins immobilization, as well as for developing potential biotechnological and environmental applications-degradation of metabolic disruptors as well [39,40,41,42]. Antibiotics used for livestock, fish farms, and humans are evolving as a serious pollutant in aquatic environments [43]. Their presence in numerous other environmental fragments including surfaces, soils, and groundwater, and some other biota is of great concern include environmental protection, antibiotic resistance, animal and human health [44,45]. Our group has reported a mechanism for thiolation of CTNs surfaces supported onto the magnetized halloysite nanotube and their application in the degradation of ampicillin and other organic compounds [46].

In this report, we propose immobilization of laccase enzyme using thiolation (–SH) mechanism of the amino (–NH_2_) groups of the CTNs supported by magnetized-α-cellulose and further employed for degradation of sulfamethoxazole a potent metabolic disruptor. The magnetized nanocomposite of CTNs supported by α-cellulose was prepared, and further thiolated the (–NH_2_) groups of CTNs, and applied effectively for immobilization laccase and degradation of the pharmaceutical compound sulfamethoxazole. Moreover, different factors including immobilization activity recovery, laccase loading capacity, the stability of pH, temperature, and storage, were first optimized. We performed the morphological and structural characterization of developed materials using independent techniques including high-resolution transmission electron microscopy (HR-TEM), X-ray diffraction (XRD), vibrating sample magnetometer (VSM), X-ray photoelectron spectroscopy (XPS), and Fourier transform infrared spectroscopy (FT-IR) analyses. The immobilized laccase enzyme in the biocompatible α-cellulose-Fe_3_O_4_-CTNs-SH-Laccase can be a novel efficient material for successfully treating water containing sulfamethoxazole antibiotics.

## 2. Materials and Methods

### 2.1. Materials

FeCl_2_·4H_2_O, FeCl_3_·6H_2_O, glutaraldehyde, and NH_3_·H_2_O were obtained from Daejung Chemicals, Sheung-si, Gyeonggi-do, South Korea. Chitosan (CTNs, low molecular weight), guaiacol (GUA, assay ≥ 98%), 2,2-Azino-bis(3 ethylbenzothiazoline-6-sulfonic acid) diammonium (ABTS, Liquid Substrate System), laccase from *Trametes Versicolor* (powder), N,N-dimethylformamide (99.8% DMF), thioglycolic acid (≥98%), N-hydroxysuccinimide (≥97.0% (titration) NHS), N-(3-dimethylaminopropyl)-N’-ethylcarbodiimide hydrochloride (≥98% (titration), EDAC.HCl), and sulfamethoxazole (SMX) were obtained by the Sigma Aldrich, St. Louis, MO, USA.

### 2.2. Synthesis

The extraction of α-cellulose from paper waste biomass, the magnetization of extracted α-cellulose (α-Cellulose-Fe_3_O_4_), and CTNs loading over magnetized α-cellulose (α-Cellulose-Fe_3_O_4_-CTNs) was carried out as previous report [33]. The thiolation of α-Cellulose-Fe_3_O_4_-CTNs was carried out in the following steps. First, α-Cellulose-Fe_3_O_4_-CTNs was washed thoroughly with distilled water to remove impurities. This well washed 100 mg of α-Cellulose-Fe_3_O_4_-CTNs was added to a 20 mL glass tube. The 15 mL of Na-acetate buffer (pH 5.0 100 mM) was added to the tube. The mixture was ultrasonicated for 15 min with power—130 W, frequency—20 kHz, and amplitude—60 µM (Sonics Vibra-Cell VC130 Ultrasonic Processor, Sonics & Materials, Inc., Newtown, CT, USA). The NHS-ester solution was prepared as per the previous report [46]. In a typical NHS-ester reaction, NHS (5.79 mM), EDAC (6.08 mM), and finally 5 mL of TGA were added to 10 mL DMF, and resulting mixture was held for shaking conditions of 200 rpm for 24 h at 25 °C. After the formation of NHS-ester, its reaction with α-Cellulose-Fe_3_O_4_-CTNs was carried out. For this, NHS-ester (0.67 mL/L) was added to ultrasonicated 15 mL solution of α-Cellulose-Fe_3_O_4_-CTNs. The mixture was kept in shaking conditions for 4 h, 25 °C, and at 200 rpm under dark conditions. After completion of this reaction, the thiolated α-Cellulose-Fe_3_O_4_-CTNs (α-Cellulose-Fe_3_O_4_-CTNs-SH) were separated magnetically. The separated α-Cellulose-Fe_3_O_4_-CTNs-SH was washed thoroughly with Na-acetate buffer (pH 5.0 100 mM) several times. The well washed α-Cellulose-Fe_3_O_4_-CTNs-SH was further applied in laccase immobilization experiments.

### 2.3. Laccase Immobilization on α-Cellulose-Fe*_3_*O*_4_*-CTNs-SH

The laccase (1.5 mg/mL) immobilization on α-Cellulose-Fe_3_O_4_-CTNs-SH (1 g/L) was carried out in a 20 mL glass tube containing 10 mL of Na-acetate buffer (pH 4.0, 100 mM). The mixture was gently mixed and incubated at shaking conditions of 200 rpm, 20 °C, and 24 h. After completion of the immobilization experiment, α-Cellulose-Fe3O4-CTNs-SH-Laccase was separated magnetically. The α-Cellulose-Fe_3_O_4_-CTNs-SH-Laccase was washed thoroughly with Na-acetate buffer (pH 4.0, 100 mM). The activity of laccase was done in a reaction mixture of 2 mL composed of substrate 1 mL of ABTS solution (90 µM), free laccase (0.1 mL from 1.5 mg/mL of laccase solution) or α-Cellulose-Fe_3_O_4_-CTNs-SH-Laccase (0.1 mL solution containing 1 mg of α-Cellulose-Fe_3_O_4_-CTNs-SH-Laccase), and 0.9 mL of sodium acetate buffer (100 mM, pH 4). The activity testing reaction was carried out at 200 rpm, 20 °C, and for 20 min. The immobilization parameters, such as activity recovery (%), relative activity (%), and laccase loading capacity (mg/g), were measured as per equations reported in earlier reports [46]. The details of these equations are given as follows. The formulae of the measurements of activity recovery (%) were given by Equation (1):

(1) $$Activity\;recovery\;\left(\%\right)=A_{IL}/A_{FL}\times 100$$

where *A_IL_* is the immobilized laccase activity, and *A_FL_* is the free laccase activity before the immobilization procedure. The laccase loading capacity on the α-Cellulose-Fe_3_O_4_-CTNs-SH (mg/g) was obtained by the Bradford method by using the Pierce™ Coomassie (Bradford) Protein Assay Kit, Thermo Scientific™, Massachusetts, MA, USA. The measurements of laccase loading capacity (mg/g) were given by Equation (2):

(2) $$Laccase\;loading\;(mg/g)=\left(C_{bi}-C_{ai}\right)V/W$$

where *C_bi_* is laccase concentration before immobilization (mg/L), *C_ai_* is retained laccase concentration in solution after immobilization (mg/L), *V* is the volume of the solution in liters (L), and *W* is the weight of α-Cellulose-Fe_3_O_4_-CTNs-SH in gram (*g*). Furthermore, the relative activity is given by the following Formulae (3):

(3) $$Relative\;activity\;\left(\%\right)=A_{e}/A_{i}\;\times 100$$

where *A_e_* is the activity after the stability experiment, and *A_i_* is the initial activity before the stability experiment. The effect of initial laccase concentrations 0.3, 0.6, 0.9, 1.2, 1.5, and 1.8 mg/mL on activity recovery (%) and laccase loading capacity (mg/g) on α-Cellulose-Fe_3_O_4_-CTNs-SH (1 g/L) was tested in Na-acetate buffer (pH 4.0, 100 mM), at 200 rpm, 20 °C, and 24 h. Further, the thermal stability of α-Cellulose-Fe3O4-CTNs-SH-Laccase was carried out by incubating α-Cellulose-Fe_3_O_4_-CTNs-SH-Laccase and free laccase at 60 °C for 180 min in Na-acetate buffer (pH 4.0, 100 mM). The relative activity (%) was tested after each 40 min interval. The temperature for testing the temperature stability was used to be 60 °C. The reason behind this is to evaluate the stability variations in the immobilized laccase than free laccase at the higher temperature. Furthermore, the pH stability study for α-Cellulose-Fe_3_O_4_-CTNs-SH-Laccase was carried out by incubating it at various pH (1–9) at 200 rpm, 20 °C, and 1 h. The relative activity (%) was tested after completion of the incubation. The storage stability study was done by incubating α-Cellulose-Fe_3_O_4_-CTNs-SH-Laccase at 4 °C for 30 days. After every 5 days, samples were tested for the relative activity (%). Finally, the re-usability experiment for α-Cellulose-Fe_3_O_4_-CTNs-SH-Laccase was done. The reactants in first cycle include; substrate ABTS 1 mL (90 µM), α-Cellulose-Fe_3_O_4_-CTNs-SH-Laccase (0.1 mL solution containing 1 mg of α-Cellulose-Fe_3_O_4_-CTNs-SH-Laccase), and 0.9 mL of sodium acetate buffer (100 mM, pH 4) solution. The first cycle reaction was carried out at 200 rpm, 20 °C, and for 20 min. After completion of the first cycle, resulting α-Cellulose-Fe_3_O_4_-CTNs-SH-Laccase was separated magnetically, added with a fresh reactant, and incubated at 200 rpm, 20 °C, and for 20 min to carry out the second cycle. Similar, 10 cycle reactions were carried out to assess the reusability potential of α-Cellulose-Fe_3_O_4_-CTNs-SH-Laccase. The details of material characterizations were given in Supplementary Materials Method S1.

### 2.4. SMX Degradation by α-Cellulose-Fe*_3_*O*_4_*-CTNs-SH-Laccase

In the typical reaction mixture, SMX (25 ppm), α-Cellulose-Fe_3_O_4_-CTNs-SH-Laccase (0.1 mL solution containing 1 mg of α-Cellulose-Fe_3_O_4_-CTNs-SH-Laccase), redox-mediator GUA (500 µM) and sodium acetate buffer (100 mM, pH 3) was taken and kept shaking at 200 rpm, 30 °C for 20 h. After completion of the reaction, α-Cellulose-Fe_3_O_4_-CTNs-SH-Laccase separated magnetically, and retained solution analyzed spectrophotometrically at 287 nm to determine SMX degradation [47]. The optimization of reaction time for SMX degradation was done studying SMX degradation at 4, 8, 12, 16, 20, and 24 h. The pH and temperature optimum for SMX degradation by α-Cellulose-Fe_3_O_4_-CTNs-SH-Laccase study was carried out by varying the initial pH of reaction from 2–8 and temperature of 20, 30, 40, and 50 °C. The effect of shaking conditions was studied by carrying out a reaction at 50, 100, 150, 200, and 250 rpm. Finally, the repeated cycle degradation of SMX was carried by α-Cellulose-Fe_3_O_4_-CTNs-SH-Laccase. In this experiment, after completion of first degradation cycle, α-Cellulose-Fe_3_O_4_-CTNs-SH-Laccase separated magnetically, and fresh reactants were added for the second cycle. Similarly, 10 degradation cycles were carried out.

### 2.5. Statistical Analysis

All the values are the average of the three experiments with ± SD (standard deviation).

## 3. Results and Discussion

### 3.1. Synthesis and Strategy

**Figure 1:**
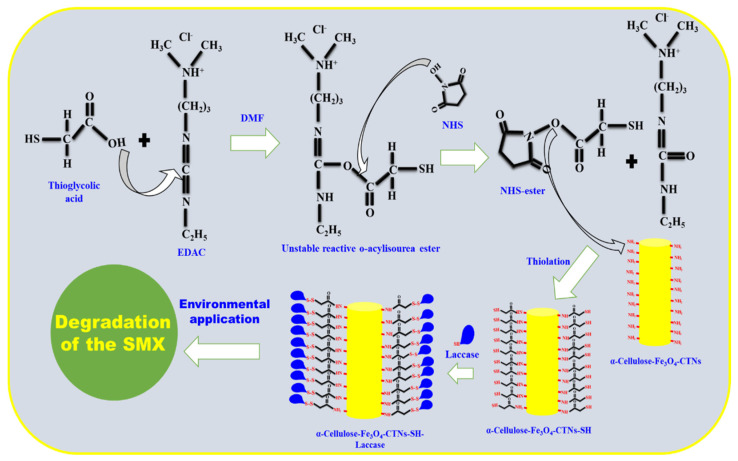
Schematic presentation of a chemical scheme for laccase immobilization on α-Cellulose-Fe_3_O_4_-CTNs-SH and its environmental application.

The typical synthesis process and strategy of the study have been elaborated in Figure 1. The α-cellulose fibers were extracted from paper-waste, magnetized and chitosan modified (α-Cellulose-Fe_3_O_4_-CTNs) [33]. In this study, thiolation of α-Cellulose-Fe_3_O_4_-CTNs was carried out (α-Cellulose-Fe_3_O_4_-CTNs-SH) for immobilization of laccase. The typical thiolation of α-Cellulose-Fe_3_O_4_-CTNs was shown in Figure 1. The thioglycolic acid and EDAC reacts to form the unstable reactive o-acylisourea ester. The NHS reacts with the unstable reactive o-acylisourea ester to form the reactive NHS-ester. The NHS-ester attacks the amino groups of the α-Cellulose-Fe_3_O_4_-CTNs, and transfer the thiol group to form α-Cellulose-Fe_3_O_4_-CTNs-SH [46]. Further, the laccase was immobilized on the α-Cellulose-Fe_3_O_4_-CTNs-SH (α-Cellulose-Fe_3_O_4_-CTNs-SH-Laccase). This developed nano-bio-catalyst α-Cellulose-Fe_3_O_4_-CTNs-SH-Laccase was further investigated the environmental application of SMX degradation (Figure 1).

### 3.2. Characterizations

#### 3.2.1. HR-TEM Analysis

**Figure 2:**
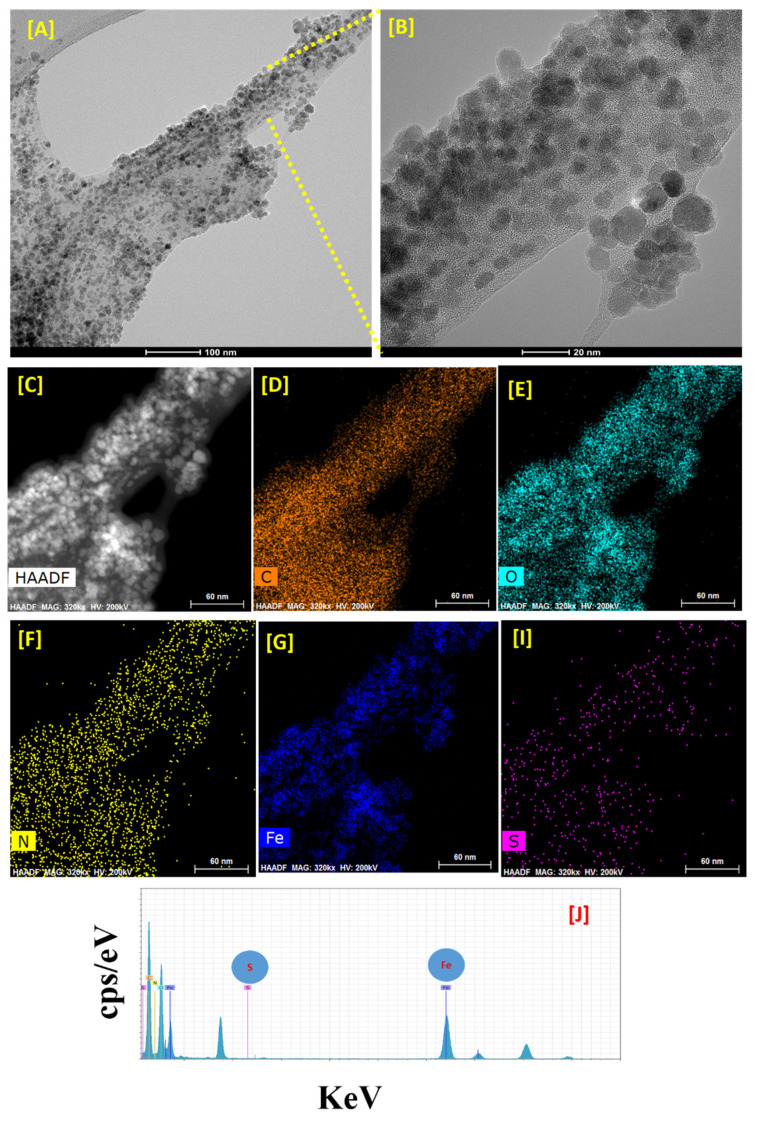
High-resolution transmission electron microscopy (HR-TEM) image of (**A**) α-Cellulose-Fe_3_O_4_-CTNs-SH, (**B**) Zoomed view of the α-Cellulose-Fe_3_O_4_-CTNs-SH HR-TEM image, (**C**) high-angle annular dark-field imaging (HAADF) analysis of the α-Cellulose-Fe_3_O_4_-CTNs-SH, HR-TEM HAADF elemental mapping (**D**) carbon (C), (**E**) oxygen (O), (**F**) nitrogen (N), (**G**) iron (Fe), and (**I**) sulfur (S), and (**J**) HR-TEM EDS profile of the α-Cellulose-Fe_3_O_4_-CTNs-SH.

Figure 2 shows HR-TEM imaging, high-angle annular dark-field imaging (HAADF)-STEM, and EDS analysis of the α-Cellulose-Fe_3_O_4_-CTNs-SH. Figure 2A represents the surface of a particular α-cellulose loaded with Fe_3_O_4_ NPs all-round the surface area. We found the Fe_3_O_4_ NPs size distribution from 4 to 12 nm range with an average of about 8 nm ± 3. The shape of Fe_3_O_4_ NPs were mostly observed for having circular to quasi-polyhedral. The magnified HR-TEM image shown in Figure 2B confirms α-cellulose effectively provides surface area and sites for nucleation and in-situ growth of Fe_3_O_4_ NPs.

The waste-paper obtained α-cellulose acted as both template and spacer to result in Fe_3_O_4_ NPs having narrow distribution, without any undesirable aggregation or agglomeration. Figure 2C also shows HAADF-STEM scan images of the carbon (Figure 2D), oxygen (Figure 2E), nitrogen (Figure 2F), Fe (Figure 2G), and sulfur (Figure 2I), for the selected area (Figure 2C). The elemental mapping results showed that the distribution of Fe and O in Fe_3_O_4_ were consistently validated. Furthermore, it was significant to reveal that the subsequent modification of the Fe_3_O_4_ loaded α-cellulose with CTNs. The presence of surface element S was clearly evident confirmation of thiolation onto the CTNs surfaces. Besides, the EDX pattern exhibited prominent peaks for iron, oxygen, carbon, and sulfur in the elemental composition of α-Cellulose-Fe_3_O_4_-CTNs-SH. This observation also confirmed the successful thiolation of α-Cellulose-Fe_3_O_4_-CTNs. As one can see in Figure 2J, α-Cellulose-Fe_3_O_4_-CTNs-SH was prepared successfully with element composition comprising, Fe (22.52%), O (18.46%), C (57.51%), N (1.05%), and S (0.04%). These all HR-TEM results confirmed that α-cellulose was successfully modified with the Fe_3_O_4_ loading, CTNs grafting, and thiolation grafted chitosan.

#### 3.2.2. XRD, VSM, XPS, and FT-IR Analysis

**Figure 3:**
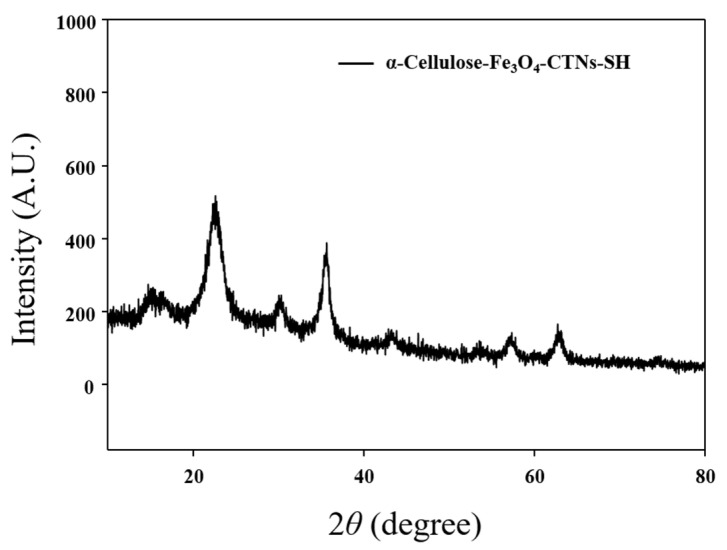
XRD analysis of the α-Cellulose-Fe_3_O_4_-CTNs-SH.

Figure 3 displays the XRD patterns of composite α-Cellulose-Fe_3_O_4_-CTNs-SH. The XRD curve was approximately similar to that of Fe_3_O_4_ NPs [48], and there was no diffraction peak belonging to sulfur at 2θ = 20 to 70°, indicating that -SH or thiol is organic and amorphous. XRD results revealed the excellent stability of Fe_3_O_4_ NPs after the thiolation process performed for the immobilization of laccase enzymes. The Fe_3_O_4_ NPs diffraction peaks were assigned at 22.57°, 35.61, 43.39, 57.09, and 62.88° 2θ angles conforming to the crystalline planes for the (111), (311), (400), (511), and (440), respectively as reported in the previous report [31]. The typical XRD peak for cellulose-I was observed at 14.76 attributed to the plane (110), this peak is in agreement with a previous report [49]. The characteristic XRD peaks specify that the α-cellulose surface was well decorated with Fe_3_O_4_ NPs, thus clear diffraction peaks appeared with strong intensity. Thus, the XRD analysis confirms successful loading and excellent stability of Fe_3_O_4_ NPs onto the α-cellulose surfaces.

**Figure 4:**
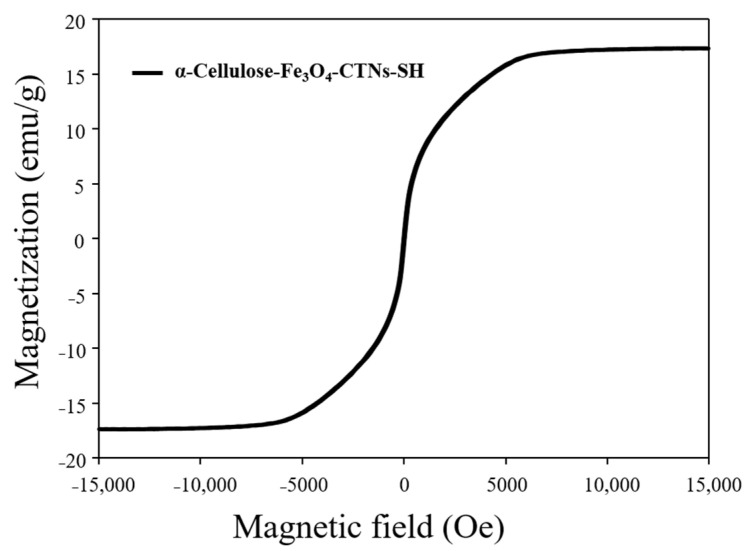
Vibrating sample magnetometer (VSM) analysis of the α-Cellulose-Fe_3_O_4_-CTNs-SH.

The magnetic properties of nano-supports developed for enzyme immobilization were highly important. As it allows to retrieve enzyme-immobilized nano-support from the reaction solution by applying external magnetic force. This removes immobilized-enzyme from the solution very easily, and it can be further applied in the next reaction or next cycle. To confirm the magnetic properties, VSM analysis of α-Cellulose-Fe_3_O_4_-CTNs-SH was performed. Figure 4 gave VSM plot of the α-Cellulose-Fe_3_O_4_-CTNs-SH. The obtained curve showed typical magnetic hysteresis with zero coercivity and remanence values. The obtained magnetic saturation value was found to be 17.38 emu/g. All these properties confirmed the super-para-magnetic nature of the α-Cellulose-Fe_3_O_4_-CTNs-SH.

**Figure 5:**
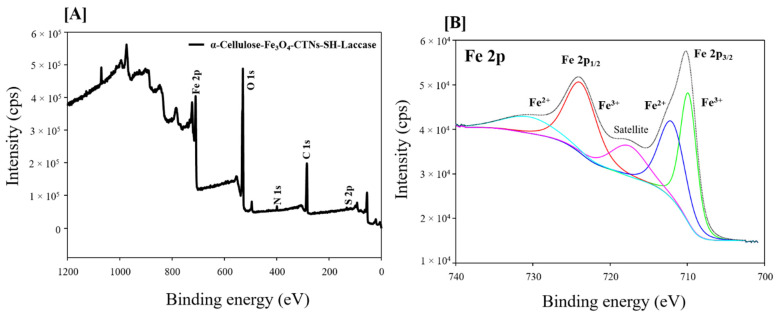
(**A**) XPS analysis of the α-Cellulose-Fe_3_O_4_-CTNs-SH-Laccase, and (**B**) high-resolution Fe 2p XPS spectrum of α-Cellulose-Fe_3_O_4_-CTNs-SH-Laccase.

XPS analysis was used to characterize the elemental profile of α-Cellulose-Fe_3_O_4_-CTNs-SH-Laccase. Figure 5A shows the XPS spectra and their peaks C 1s, N 1s, O 1s, and Fe 2p, at binding energies (BEs) of 284.6, 399.70, 529.52, and 710.0 eV, respectively. Among them, peak at 399.7 eV assigns to -NH_2_ of The CTNs and laccase. XPS spectra and elements confirm successful loading of the presence of Fe_3_O_4_ NPs and CTNs. Furthermore, the high-resolution spectra of Fe 2p were performed for α-Cellulose-Fe_3_O_4_-CTNs-SH-Laccase by curve fitting analysis (Figure 5B). One can see the curve fittings results obtained for Fe 2p shows two basic peaks of Fe 2p_3/2_ and Fe 2p_1/2_ at the BEs of 724.17 and 710.16 eV, respectively. The spin energy separation of Fe 2p_3/2_ and Fe 2p_1/2_ was found to be 14.01 eV, which in agreement with the previous report on Fe_3_O_4_ NPs [50]. Since Fe 2p curve fitting analysis indicated the presence of both the oxidation states of Fe^2+^ and Fe^3+^ [51]. The XPS results revealed that Fe_3_O_4_ was remained stable onto the α-cellulose ever after modified using CTNs and laccase immobilization.

**Figure 6:**
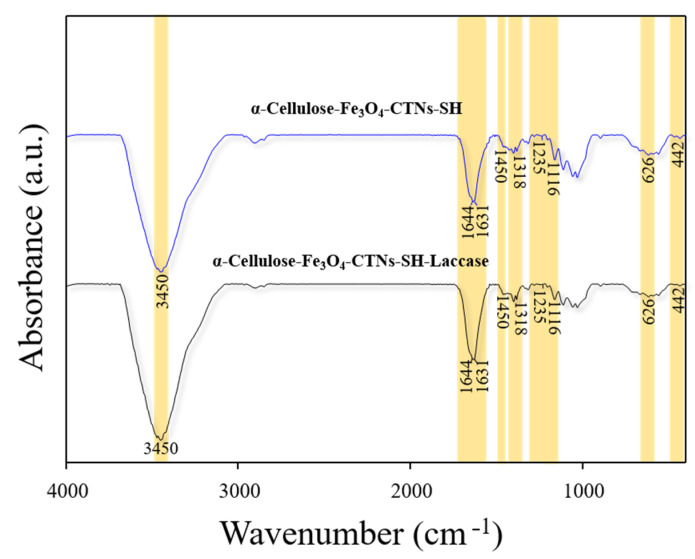
FT-IR analysis of the α-Cellulose-Fe_3_O_4_-CTN-SH and α-Cellulose-Fe_3_O_4_-CTN-SH-Laccase.

The surface functional groups of α-Cellulose-Fe_3_O_4_-CTNs-SH and α-Cellulose-Fe_3_O_4_-CTNs-SH-Laccase biocatalyst was examined by the FT-IR analysis, shown in Figure 6. The absorption peak observed at 442 cm^−1^ attributes to the Fe–O bond results from Fe_3_O_4_ NPs [33]. The absorption peaks of 1116, 1318, 1450, 1631, and 3450 cm^−1^ obtained in all the samples, corresponds to the C–OH stretching, C–N vibrations, C–H deformation, N–H deformation, O–H stretching vibrations either from α-cellulose or CTNs as reported previously [46]. The α-cellulose materials consist of three types of atoms, carbon, oxygen, and hydrogen, which creates a straight-chain biopolymer having OH groups, and glucose rings. However, both the samples resulted in a similar peak profile consistent to the α-Cellulose-Fe_3_O_4_-CTNs-SH and α-Cellulose-Fe_3_O_4_-CTNs-SH-Laccase. However, samples after laccase enzyme immobilization resulted in FTIR peak at 1644 cm^−1^ (amide I) with absorption intensity owing to successful laccase enzyme immobilization. However, prominent FTIR peaks were observed for α-Cellulose-Fe_3_O_4_-CTNs-SH and α-Cellulose-Fe_3_O_4_-CTNs-SH-Laccase at 1644, 1235, and 626 cm^−1^ corresponds to the stretching vibrations from amides, C–SH stretching, and C–S stretching from thiol, respectively [52,53]. The appearance of the C–SH stretching peak effectively validates the thiolation of the CTNs. However, as the C–S stretching appeared in both samples, the distinctive FT-IR absorption peak for –S-S– (disulfide bond) was not observed in α-Cellulose-Fe_3_O_4_-CTNs-SH-Laccase. This might be due to the complex nature of nano-composite or overcrowding of the laccase over the surface of the α-Cellulose-Fe_3_O_4_-CTNs-SH. We found the FTIR spectral analysis in good agreement with HR-TEM, HAADF-STEM, and EDS elemental mapping results.

### 3.3. Laccase Immobilization Studies

#### 3.3.1. Activity Recovery and Laccase Loading Capacity Evaluations

**Figure 7:**
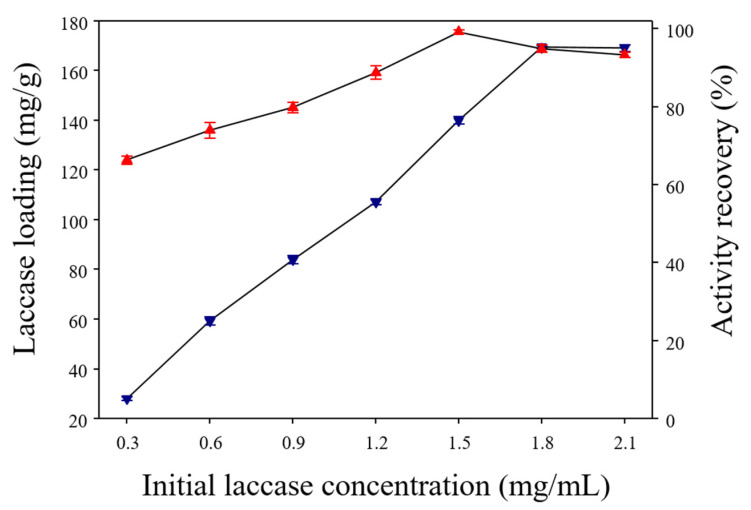
Laccase loading pattern on α-Cellulose-Fe_3_O_4_-CTNs-SH, and laccase activity recovery by α-Cellulose-Fe_3_O_4_-CTNs-SH-Laccase.

The laccase loading pattern and subsequent activity recoveries with increasing initial laccase concentrations were shown in Figure 7. The laccase immobilized on α-Cellulose-Fe_3_O_4_-CTNs-SH gave laccase loading capacities of 27.87, 59.87, 83.76, 107.04, 139.89, 169.36, and 169.0 mg/g at initial laccase concentrations of 0.3, 0.6, 0.9, 1.2, 1.5, 1.8, and 2.1 mg/mL, respectively. Initially, with an increase in laccase concentration, the loading capacity was enhanced. At the concentration of laccase (1.8 mg/mL), loading reached a peak and remained constant further increase in the initial laccase concentration. This might be due to occupying all the immobilization sites to reach the confluence. Simultaneously, the activity recoveries of immobilized laccase was increased with an increase in initial laccase concentrations (Figure 7). The laccase activity recoveries of α-Cellulose-Fe_3_O_4_-CTNs-SH with initial laccase concentrations of 0.3, 0.6, 0.9, 1.2, 1.5, 1.8, and 2.1 mg/mL, were found to be 66.22, 73.85, 79.76, 88.69, 99.16, 94.86, and 93.28%, respectively (Figure 7). In comparison, the activity recoveries increase with an increase in the laccase loading capacity until the concentration of the 1.5 mg/mL (Figure 7). The higher enzyme activity recovery by immobilization support mainly defends upon the optimum active enzyme loading. Sometimes, a further increase in the enzyme loading causes crowding of the enzyme and results in a decrease in activity recovery. Thus, nano-support α-Cellulose-Fe_3_O_4_-CTNs-SH showed higher activity recovery with an initial laccase concentration of 1.5 mg/mL. This suggests that further increase in initial laccase concentration from the 1.5 to 1.8 mg/mL, laccase loading increases, while activity recovery remains constant. This might be due to overloading or crowding of the laccase over the surface of α-Cellulose-Fe_3_O_4_-CTNs-SH. Hence, the detailed activity recovery and laccase loading capacity data provided the information about immobilization behavior of α-Cellulose-Fe_3_O_4_-CTNs-SH. The significant laccase loading obtained by α-Cellulose-Fe_3_O_4_-CTNs-SH was quite high among the recently reported materials [33,46,54,55,56,57,58,59,60,61]. Thus, the developed α-cellulose based material has significant potential to look like an enzyme immobilization material.

#### 3.3.2. Stability Studies of Immobilized Laccase

**Figure 8:**
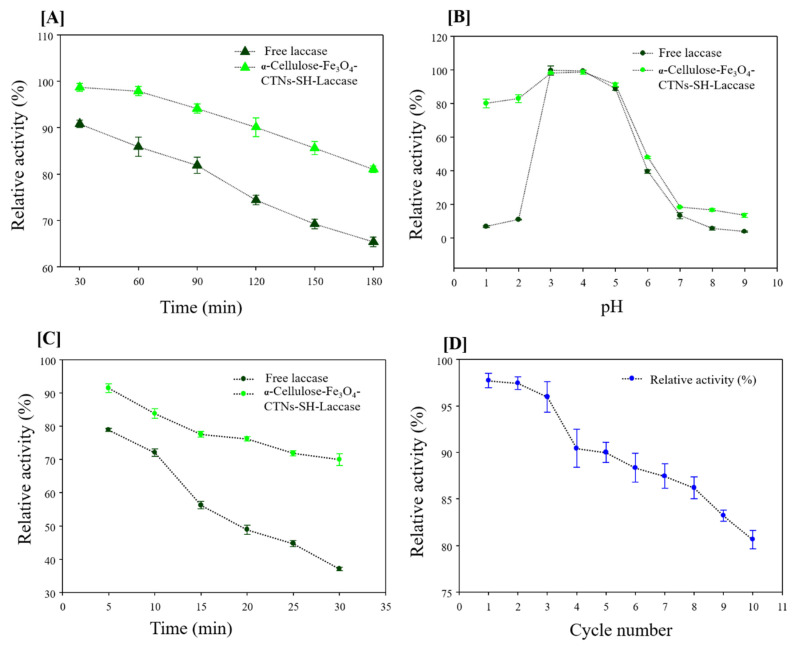
(**A**) Temperature stability of free and α-Cellulose-Fe_3_O_4_-CTNs-SH-Laccase carried out at 60 °C for 180 min time, (**B**) pH stability study of free and α-Cellulose-Fe_3_O_4_-CTNs-SH-Laccase carried out at pH 1–9 for 1 h incubation time, (**C**) storage stability study of free and α-Cellulose-Fe_3_O_4_-CTNs-SH-Laccase carried out at 4 °C and for 30 days, and (**D**) reusability study of the α-Cellulose-Fe_3_O_4_-CTNs-SH-Laccase in 10 cycles.

The immobilization strategy provides several advancements for enzyme biocatalysis [34,37]. This mainly includes temperature stability, pH stability, and storage stability compared to the free laccase. The temperature stability of α-Cellulose-Fe_3_O_4_-CTNs-SH-Laccase was explained in Figure 8A. The α-Cellulose-Fe_3_O_4_-CTNs-SH-Laccase exhibited relative activity (%) of 98, 97, 94, 90, 85, and 81%, at 30, 60, 90, 120, 150, and 180 min of the incubation at temperature 60 °C, respectively (Figure 8A). However, free laccase possessed 90, 85, 81, 74, 69, and 65% of relative activity, at 30, 60, 90, 120, 150, and 180 min of the incubation at 60 °C temperature. The obtained data suggest that the thermal stability of immobilized laccase enhanced significantly. The binding of the enzyme to support provides additional support for the enzyme to enhance thermal stabilities [37].

Further, the stability of free and immobilized laccase was assessed at various pH (Figure 8B). At the incubation pH of 1, 2, 3, 4, 5, 6, 7, 8, and 9, free laccase possessed relative activities of the 6, 10, 99, 99, 88, 39, 13, 5, and 3%, and immobilized laccase shown relative activities of 80, 82, 97, 98, 91, 47, 18, 16, and 13%, respectively (Figure 8B). These results depict enhanced pH stabilities. Particularly, at acidic pH immobilized laccase performed excellent catalysis compared to the free laccase. It is well known that the pH of the reaction is a very curtail parameter for the enzymatic reaction, hence enhancing pH stabilities at pH range (1–9) by immobilized laccase will open new facets of applications that can be exploited and studied. Thus, the α-Cellulose-Fe_3_O_4_-CTNs-SH-Laccase exhibited excellent pH stability at 1–2, and hence this immobilized system can be applied in the particular field of laccase application where acidic pH is mandatory. Similar, results of higher low pH stability obtained by previous reports [33]. The higher activity observed at the lower pH by immobilization might be due to the enhanced lower pH stability by multipoint attachment of laccase with α-Cellulose-Fe_3_O_4_-CTNs-SH. Additionally, the microenvironment of α-Cellulose-Fe_3_O_4_-CTNs-SH-Laccase and bulk-solution typically takes unequal distributions of H^+^ and OH^−^ ion-concentrations, due to their electrostatic interactions with nano-support. This mostly results in the displacements in the pH activity profile of free and immobilized laccase.

Moreover, the storage of free enzyme solution is also one of the major concerns [38]. Long-term storage will enhance the applicability and cost-effectiveness of the biocatalyst [39]. The free and immobilized laccase was tested for storage stability by incubating it at 4 °C for 30 days. The obtained results are displayed in Figure 8C. The free laccase gave relative activity of the 78, 72, 56, 48, 44, and 37% and immobilized laccase gave 91, 83, 77, 76, 71, and 68% relative activity, at the 5, 10, 15, 20, 25, and 30 days incubation, respectively. Thus, the obtained data revealed that the α-Cellulose-Fe_3_O_4_-CTNs-SH-Laccase displayed improved storage stability over free laccase. Therefore, all the stability analysis (Figure 8A–C) of α-Cellulose-Fe_3_O_4_-CTNs-SH-Laccase corroborated better biocatalysis than free laccase. Being immobilization material developed from paper-waste material and having excellent biocatalysis performance, this will be an excellent and valuable strategy to convert waste material to the best material.

#### 3.3.3. Reusability Studies of Immobilized Laccase

The industrial application of free enzymes is mostly possible in one attempt only, as the separation of products, reactant, and enzyme from solution extremely cost-effective task [38]. Hence, immobilizing the enzyme on nano-support with excellent magnetic property is significantly valuable to apply enzyme-biocatalysis in multiple cycles. Hence, in this study, laccase was immobilized on super-magnetic α-Cellulose-Fe_3_O_4_-CTNs-SH and applied in the 10 repeated cycles (Figure 8D). The α-Cellulose-Fe_3_O_4_-CTNs-SH-Laccase gave 97, 97, 95, 90, 89, 88, 87, 86, 83, and 80% relative activity. The obtained reusability results are highly encouraging and important to apply laccase biocatalysis for various applications. Thus, all these biocatalysis studies confirmed the potential of the α-Cellulose-Fe_3_O_4_-CTNs-SH-Laccase as a novel, cost-effective, and environmental-friendly biocatalyst for laccase based applications.

By considering the valuable materials like α-Cellulose-Fe_3_O_4_-CTNs, and its application in the development of new enzyme immobilization techniques were highly essential. In previous work, laccase immobilization on α-Cellulose-Fe_3_O_4_-CTNs was done by cross-linking agent glutaraldehyde (GTA) [33]. Therefore, we compared the obtained results of this study with “GTA modified α-Cellulose-Fe_3_O_4_-CTNs” for laccase immobilization. The thiol modified α-Cellulose-Fe_3_O_4_-CTNs (α-Cellulose-Fe_3_O_4_-CTNs-SH) gave a significantly higher capacity of the laccase loading (169 mg/g) than the GTA modified α-Cellulose-Fe_3_O_4_-CTNs (73.30 mg/g) [33]. Similarly, the stability of temperature was improved with α-Cellulose-Fe3O4-CTNs-SH-Laccase [33]. Looking at the obtained results, thiol modified α-Cellulose-Fe_3_O_4_-CTNs were found to be an excellent material for enzyme immobilization strategy.

### 3.4. Degradation of SMX by α-Cellulose-Fe_3_O_4_-CTNs-SH-Laccase

**Figure 9:**
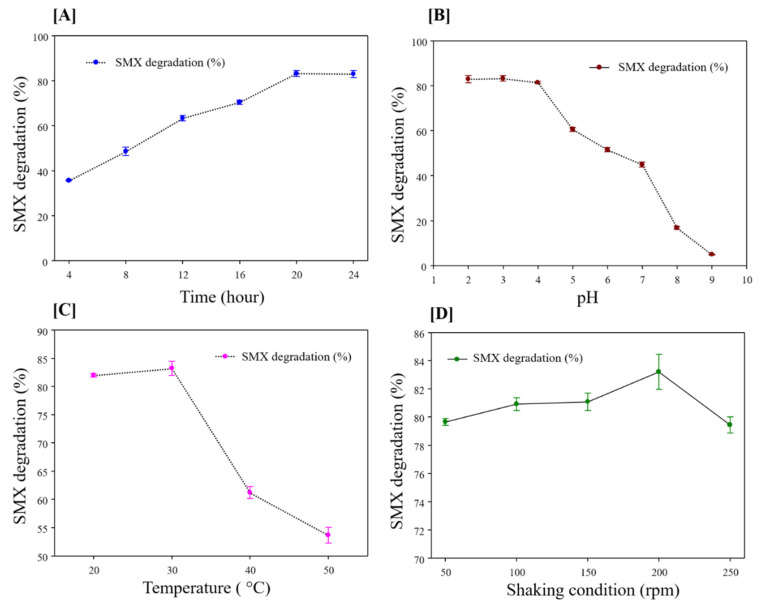
Effect of time (**A**), pH (**B**), temperatures (**C**), and shaking conditions (**D**) on SMX degradation by the α-Cellulose-Fe_3_O_4_-CTNs-SH-Laccase.

Laccase is a well-known biocatalyst for the degradation of environmental pollutants [60,61]. The pharmaceutical compound “SMX” causes serious environmental and health issues [61]. Hence, in this study immobilized laccase was tested for the degradation of SMX. The degradation of SMX by α-Cellulose-Fe_3_O_4_-CTNs-SH-Laccase was assessed for incubation time 4 to 24 h (Figure 9A). At the 4, 8, 12, 16, 20, and 24 h, α-Cellulose-Fe_3_O_4_-CTNs-SH-Laccase gave 35, 48, 63, 70, 83, and 82% degradation of the SMX, respectively (Figure 9A). The increase in incubation time of SMX with α-Cellulose-Fe_3_O_4_-CTNs-SH-Laccase increases the degradation of SMX until 20 h. After 20 h, SMX degradation remains constant. Hence, the optimum time for SMX degradation was found to be 20 h. Further, the effect of pH on SMX degradation was evaluated (Figure 9B). At the pH of 2, 3, 4, 5, 6, 7, 8, and 9, α-Cellulose-Fe_3_O_4_-CTNs-SH-Laccase gave 82, 83, 81, 60, 51, 44, 16, and 5% degradation, respectively. The higher SMX degradation was observed at the acidic pH range of 2–4. After understanding the effect of pH, the effect of temperature was assessed for α-Cellulose-Fe_3_O_4_-CTNs-SH-Laccase mediated degradation of the SMX (Figure 9C). At the temperatures of 20, 30, 40, and 50, SMX degradation of 81, 83, 61, and 50 was observed. The higher SMX degradation was observed at 30 °C. Moreover, the effect of shaking conditions on the SMX degradation by α-Cellulose-Fe_3_O_4_-CTNs-SH-Laccase was displayed in (Figure 9D). The shaking conditions of 50, 100, 150, 200, and 250 rpm yielded 79, 80, 81, 83, and 79% SMX degradation. The higher degradation was observed at 200 rpm. However, not significant difference or effect of shaking condition was observed on the degradation of SMX by α-Cellulose-Fe_3_O_4_-CTNs-SH-Laccase. Thus, all the optimized parameters; incubation time, pH, temperatures, and shaking conditions were highly important to scale-up of the treatment protocol for α-Cellulose-Fe_3_O_4_-CTNs-SH-Laccase mediated SMX degradation.

**Figure 10:**
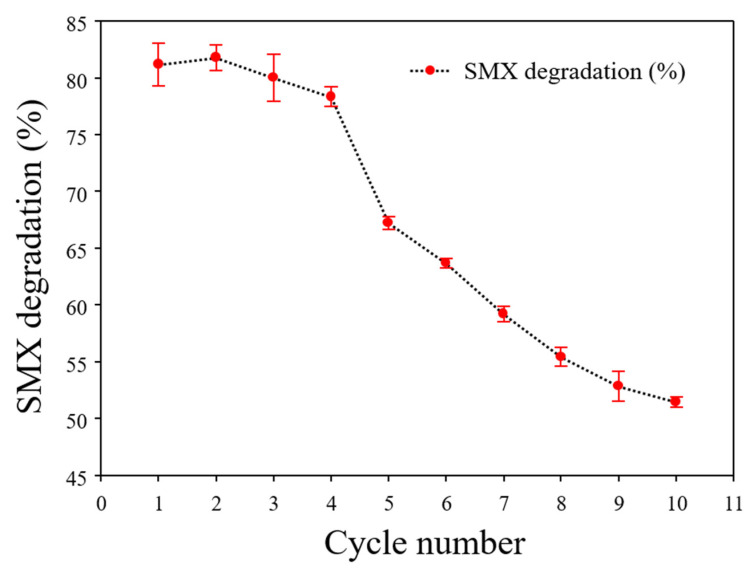
The repeated cycle degradation of SMX by α-Cellulose-Fe_3_O_4_-CTNs-SH-Laccase.

Furthermore, the repeated degradation of SMX by α-Cellulose-Fe3O4-CTNs-SH-Laccase was performed in a repeated cycle experiment (Figure 10). The free laccase can access its application of SMX degradation in only one cycle. After completion of the first degradation cycle, α-Cellulose-Fe3O4-CTNs-SH-Laccase was retrieved by an external magnet, washed thoroughly with acetate buffer solution (100 mM, pH 3), and added with the fresh reactants. Such 10 number of cycles yielded 81, 81, 80, 78, 67, 63, 59, 55, 52, and 51% of the SMX degradation. At the end of the 10th cycle, α-Cellulose-Fe_3_O_4_-CTNs-SH-Laccase exhibited 51% of SMX degradation. These all results corroborated the potential of α-Cellulose-Fe_3_O_4_-CTNs-SH-Laccase biocatalysts for environmental application and possibilities of developing a reactor scale treatment approach for decontamination of the pharmaceuticals contaminants. Thus, all biocatalysis and SMX degradation studies marked the importance of α-Cellulose-Fe_3_O_4_-CTNs-SH-Laccase catalysis.

## 4. Conclusions

In conclusion, this study developed a new immobilization nano-support using nano-bio-composite of paper-waste derived α-cellulose, Fe_3_O_4_ NPs, CTNs, and thiol (-SH) functionalization. The studied characterizations confirmed the successful synthesis of the α-Cellulose-Fe_3_O_4_-CTNs-SH-Laccase. The α-Cellulose-Fe_3_O_4_-CTNs-SH-Laccase was found to be an excellent biocatalyst with improved stabilities of thermal, pH, and storage. The reusability potential of α-Cellulose-Fe_3_O_4_-CTNs-SH-Laccase assured its potential for many laccase based applications. The significant potential of α-Cellulose-Fe_3_O_4_-CTNs-SH-Laccase was found in the decontamination of SMX from water. Thus, the developed bio-catalyst α-Cellulose-Fe_3_O_4_-CTNs-SH-Laccase is novel, super-magnetic, waste-derived, and can be applied in the degradation of environmental pollutants.

## Data Availability

Data is contained within the article.

## Supplementary Materials

The following are available online at https://www.mdpi.com/2073-4360/13/4/581/s1, Methods S1: Characterizations

## References

[1] Ma Y., Hummel M., Määttänen M., Särkilahti A., Harlin A., Sixta H. (2016). Upcycling of waste paper and cardboard to textiles. Green Chem..

[2] Zheng B., Hu C., Guan L., Gu J., Guo H., Zhang W. (2019). Structural Characterization and Analysis of High-Strength Laminated Composites from Recycled Newspaper and HDPE. Polymers.

[3] Carbone K., Paliotta M., Micheli L., Mazzuca C., Cacciotti I., Nocente F., Ciampa A., Dell’Abate M.T. (2019). A completely green approach to the synthesis of dendritic silver nanostructures starting from white grape pomace as a potential nanofactory. Arab. J. Chem..

[4] Jiang B., Na J., Wang L., Li D., Liu C., Feng Z. (2019). Reutilization of Food Waste: One-Step Extration, Purification and Characterization of Ovalbumin from Salted Egg White by Aqueous Two-Phase Flotation. Foods.

[5] Scott K., Giesekam J., Barrett J., Owen A. (2019). Bridging the climate mitigation gap with economy-wide material productivity. J. Ind. Ecol..

[6] Zabielskis P. (2014). Environmental Problems in China: Issues and Prospects. Social Issues in China.

[7] CEPI (2013). Key Statistics. European Pulp and Paper Industry.

[8] Kostag M., Jedvert K., Malek N. (2020). Cellulose Regeneration and Chemical Recycling: Closing the “Cellulose Gap” Using Environmentally Benign Solvents. Macromol. Mater. Eng..

[9] Ma Y., Rosson L., Wang X., Byrne N. (2020). Upcycling of waste textiles into regenerated cellulose fibres: Impact of pretreatments. J. Text. Inst..

[10] Rana S., Pichandi S., Parveen S., Fangueiro R. (2014). Regenerated Cellulosic Fibers and Their Implications on Sustainability. Roadmap to Sustainable Textiles and Clothing, Textile Science and Clothing Technology.

[11] Feng A., Zhang Y. (2015). Study on moisture absorption and sweat discharge of honeycomb polyester fiber. IOP Conf. Ser. Mater. Sci. Eng..

[12] Adusumali R.-B., Reifferscheid M., Weber H., Roeder T., Sixta H., Gindl W. (2006). Mechanical Properties of Regenerated Cellulose Fibres for Composites. Macromol. Symp..

[13] Seisl S., Hengstmann R., Matthes A., Beyer K., Cebulla H., Arnold M.G., Schumann A. (2021). Manmade Cellulosic Fibers (MMCF)—A Historical Introduction and Existing Solutions to a MoreSustainable Production. Sustainable Textile and Fashion Value Chains: Drivers, Concepts, Theories and Solutions.

[14] Osong S.H., Norgren S., Engstrand P. (2016). Processing of wood-based microfibrillated cellulose and nanofibrillated cellulose, and applications relating to papermaking: A review. Cellulose.

[15] Sorieul M., Dickson A., Hill S.J., Pearson H. (2016). Plant Fibre: Molecular Structure and Biomechanical Properties, of a Complex Living Material, Influencing Its Deconstruction towards a Biobased Composite. Materials.

[16] Yan L., Kasal B., Huang L. (2016). A review of recent research on the use of cellulosic fibres, their fibre fabric reinforced cementitious, geo-polymer and polymer composites in civil engineering. Compos. Part B Eng..

[17] Ramamoorthy S.K., Skrifvars M., Persson A. (2015). A Review of Natural Fibers Used in Biocomposites: Plant, Animal and Regenerated Cellulose Fibers. Polym. Rev..

[18] Cacciotti I., Mori S., Cherubini V., Nanni F. (2018). Eco-sustainable systems based on poly (lactic acid), diatomite and coffee grounds extract for food packaging. Int. J. Biol. Macromol..

[19] Delgado-Aguilar M., Tarrés Q., Pèlach M.À., Mutjé P., Fullana-i-Palmer P. (2015). Are Cellulose Nanofibers a Solution for a More Circular Economy of Paper Products?. Environ. Sci. Technol..

[20] Ellen MacArthur Foundation (2012). Towards the Circular Economy: An Economic and Business Rationale for an Accelerated Transition.

[21] Jiang B., Wang M., Wang X., Wu S., Li D., Liu C., Feng Z., Li J. (2021). Effective separation of prolyl endopeptidase from *Aspergillus Niger* by aqueous two phase system and its characterization and application. Int. J. Biol. Macromol..

[22] Jiang B., Wang L., Wang M., Wu S., Wang X., Li D., Liu C., Feng Z., Chi Y. (2021). Direct separation and purification of α-lactalbumin from cow milk whey by aqueous two-phase flotation of thermo-sensitive polymer/phosphate. J. Sci. Food Agric..

[23] Jiang B., Wang X., Wang L., Wu S., Li D., Liu C., Feng Z. (2020). Fabrication and Characterization of a Microemulsion Stabilized by Integrated Phosvitin and Gallic Acid. J. Agric. Food Chem..

[24] Allwood J.M., Ashby M.F., Gutowski T.G., Worrell E. (2011). Material efficiency: A white paper. Resour. Conserv. Recycl..

[25] (2003). Fibers, Regenerated Cellulose. Kirk-Othmer Encyclopedia of Chemical Technology.

[26] Reddy N., Yang Y. (2015). Introduction to Regenerated Cellulose Fibers. Innovative Biofibers from Renewable Resources, Reddy, N., Yang, Y., Eds..

[27] Guo X., Jiang Z., Li H., Li W. (2015). Production of recycled cellulose fibers from waste paper via ultrasonic wave processing. J. Appl. Polym. Sci..

[28] Olejnik K., Skalski B., Stanislawska A., Wysocka-Robak A. (2017). Swelling properties and generation of cellulose fines originating from bleached kraft pulp refined under different operating conditions. Cellulose.

[29] Hubbe M., Venditti R., Rojas O. (2007). What happens to cellulosic fibers during papermaking and recycling? A Review. BioResources.

[30] Reh L. (2013). Process engineering in circular economy. Particuology.

[31] Kadam A.A., Lone S., Shinde S., Yang J., Saratale R.G., Saratale G.D., Sung J.-S., Kim D.Y., Ghodake G. (2019). Treatment of Hazardous Engineered Nanomaterials by Supermagnetized α-Cellulose Fibers of Renewable Paper-Waste Origin. ACS Sustain. Chem. Eng..

[32] Kadam A., Saratale R.G., Shinde S., Yang J., Hwang K., Mistry B., Saratale G.D., Lone S., Kim D.-Y., Sung J.-S. (2019). Adsorptive remediation of cobalt oxide nanoparticles by magnetized α-cellulose fibers from waste paper biomass. Bioresour. Technol..

[33] Ghodake G.S., Yang J., Shinde S.S., Mistry B.M., Kim D.-Y., Sung J.-S., Kadam A.A. (2018). Paper waste extracted α-cellulose fibers super-magnetized and chitosan-functionalized for covalent laccase immobilization. Bioresour. Technol..

[34] Wahab R.A., Elias N., Abdullah F., Ghoshal S.K. (2020). On the taught new tricks of enzymes immobilization: An all-inclusive overview. React. Funct. Polym..

[35] Krishnamoorthi S., Banerjee A., Roychoudhury A. (2015). Immobilized enzyme technology: Potentiality and prospects. J. Enzymol. Metab..

[36] Long J., Li X., Liu X., Jin Z., Xie Z., Xu X., Lu C. (2021). Preparation of Streptavidin-Coated Magnetic Nanoparticles for Specific Immobilization of Enzymes with High Activity and Enhanced Stability. Ind. Eng. Chem. Res..

[37] Liese A., Hilterhaus L. (2013). Evaluation of immobilized enzymes for industrial applications. Chem. Soc. Rev..

[38] DiCosimo R., McAuliffe J., Poulose A.J., Bohlmann G. (2013). Industrial use of immobilized enzymes. Chem. Soc. Rev..

[39] Verma M., Kumar S., Das A., Singh J., Chamundeeswari m. (2019). Enzyme Immobilization on Chitin and Chitosan-Based Supports for Biotechnological Applications. Sustainable Agriculture Reviews 35.

[40] Grazú V., Abian O., Mateo C., Batista-Viera F., Fernández-Lafuente R., Guisán J.M. (2005). Stabilization of enzymes by multipoint immobilization of thiolated proteins on new epoxy-thiol supports. Biotechnol. Bioeng..

[41] Rusmini F., Zhong Z., Feijen J. (2007). Protein Immobilization Strategies for Protein Biochips. Biomacromolecules.

[42] Federer C., Kurpiers M., Bernkop-Schnürch A. (2021). Thiolated Chitosans: A Multi-talented Class of Polymers for Various Applications. Biomacromolecules.

[43] Hughes S.R., Kay P., Brown L.E. (2013). Global Synthesis and Critical Evaluation of Pharmaceutical Data Sets Collected from River Systems. Environ. Sci. Technol..

[44] Daughton C.G., Ruhoy I.S. (2009). Environmental footprint of pharmaceuticals: The significance of factors beyond direct excretion to sewers. Environ. Toxicol. Chem..

[45] Hernández-Pérez A., Noonin C., Söderhäll K., Söderhäll I. (2020). Environmental concentrations of sulfamethoxazole increase crayfish Pacifastacus leniusculus susceptibility to White Spot Syndrome Virus. Fish Shellfish Immunol..

[46] Kadam A.A., Sharma B., Shinde S.K., Ghodake G.S., Saratale G.D., Saratale R.G., Kim D.-Y., Sung J.-S. (2020). Thiolation of Chitosan Loaded over Super-Magnetic Halloysite Nanotubes for Enhanced Laccase Immobilization. Nanomaterials.

[47] Sifuna F.W., Orata F., Okello V., Jemutai-Kimosop S. (2016). Comparative studies in electrochemical degradation of sulfamethoxazole and diclofenac in water by using various electrodes and phosphate and sulfate supporting electrolytes. Environ. Sci. Health A.

[48] Bandi S., Hastak V., Pavithra C.L.P., Kashyap S., Singh D.K., Luqman S., Peshwe D.R., Srivastav A.K. (2019). Graphene/chitosan-functionalized iron oxide nanoparticles for biomedical applications. J. Mater. Res..

[49] Gong J., Li J., Xu J., Xiang Z., Mo L. (2017). Research on cellulose nanocrystals produced from cellulose sources with various polymorphs. RSC Adv..

[50] Sharma B., Kadam A.A., Sung J.-S., Myung J.-h. (2020). Surface tuning of halloysite nanotubes with Fe3O4 and 3-D MnO2 nanoflakes for highly selective and sensitive acetone gas sensing. Ceram. Int..

[51] Wan L., Yan D., Xu X., Li J., Lu T., Gao Y., Yao Y., Pan L. (2018). Self-assembled 3D flower-like Fe3O4/C architecture with superior lithium ion storage performance. J. Mater. Chem. A.

[52] Ulu A., Birhanli E., Boran F., Köytepe S., Yesilada O., Ateş B. (2020). Laccase-conjugated thiolated chitosan-Fe3O4 hybrid composite for biocatalytic degradation of organic dyes. Int. J. Biol. Macromol.

[53] Chauhan K., Singh P., Singhal R.K. (2015). New Chitosan–Thiomer: An Efficient Colorimetric Sensor and Effective Sorbent for Mercury at Ultralow Concentration. ACS Appl. Mater. Interfaces.

[54] Qiu X., Wang Y., Xue Y., Li W., Hu Y. (2020). Laccase immobilized on magnetic nanoparticles modified by amino-functionalized ionic liquid via dialdehyde starch for phenolic compounds biodegradation. Chem. Eng. J..

[55] Qiu X., Wang S., Miao S., Suo H., Xu H., Hu Y. (2020). Co-immobilization of laccase and ABTS onto amino-functionalized ionic liquid-modified magnetic chitosan nanoparticles for pollutants removal. J. Hazard. Mater..

[56] Zhang Y., Piao M., He L., Yao L., Piao T., Liu Z., Piao Y. (2020). Immobilization of laccase on magnetically separable biochar for highly efficient removal of bisphenol A in water. RSC Adv..

[57] Zhang K., Yang W., Liu Y., Zhang K., Chen Y., Yin X. (2020). Laccase immobilized on chitosan-coated Fe3O4 nanoparticles as reusable biocatalyst for degradation of chlorophenol. J. Mol. Struct..

[58] Jankowska K., Ciesielczyk F., Bachosz K., Zdarta J., Kaczorek E., Jesionowski T. (2019). Laccase immobilized onto zirconia-silica hybrid doped with Cu2+ as an effective biocatalytic system for decolorization of dyes. Materials.

[59] Cao P., Liu H., Wu D., Wang X. (2020). Immobilization of laccase on phase-change microcapsules as selfthermoregulatory enzyme carrier for biocatalytic enhancement. Chem. Eng. J..

[60] Kadam A.A., Jang J., Jee S.C., Sung J.S., Lee D.S. (2018). Chitosan-functionalized supermagnetic halloysite nanotubes for covalent laccase immobilization. Carbohydr. Polym..

[61] Kadam A.A., Shinde S.K., Ghodake G.S., Saratale G.D., Saratale R.G., Sharma B., Hyun S., Sung J.-S. (2020). Chitosan-Grafted Halloysite Nanotubes-Fe3O4 Composite for Laccase-Immobilization and Sulfamethoxazole-Degradation. Polymers.

