# Supplementary material for: α-Cellulose Fibers of Paper-Waste Origin Surface-Modified with Fe3O4 and Thiolated-Chitosan for Efficacious Immobilization of Laccase
Source: Polymers (Basel). 2021 Feb 15;13(4):581. doi: 10.3390/polym13040581 (PMC7919293; doi:10.3390/polym13040581)
Supplement: Supplementary file 1 [file polymers-13-00581-s001.pdf]

Supplementary information

α-Cellulose fibers of paper-waste origin surface-modified with Fe3O4 and thiolated-chitosan for efficacious immobilization of laccase

Gajanan S. Ghodake[1], Surendra K. Shinde[1], Ganesh D. Saratale[2], Rijuta, G. Saratale[3], Min Kim[4], Seung-Cheol Jee[4], Dae-Young Kim[1], Jung-Suk Sung[4], and Avinash A. Kadam[3,*]

[1] Department of Biological and Environmental Science, Dongguk University-Seoul, Ilsandong-gu, Goyang-si, Gyonggido, Seoul 10326, Korea; ghodakegs@gmail.com (G.S.G.); surendrashinde.phy@gmail.com (S.K.S.), sbpkim@dongguk.edu (D.-Y.K.)

[2] Department of Food Science and Biotechnology, Dongguk University-Seoul, Ilsandong-gu, Goyang-si, Gyeonggi-do, Seoul 10326, Korea; gdsaratale@gmail.com (G.D.S.)

[3] Research Institute of Biotechnology and Medical Converged Science, Dongguk University-Seoul, Ilsandong-gu, Goyang-si, Gyeonggi-do, Seoul 10326, Korea. rijutaganesh@gmail.com (R.G.S.); kadamavinash@dongguk.edu (A.A.K.)

[4] Department of Life Science, College of Life Science and Biotechnology, Dongguk University-Seoul, Ilsandong-gu, Goyang-si, Gyonggido, Seoul 10326, Korea. pipikimmin@naver.com (M.K.); markjee@naver.com (S.-C.J.); sungjs@dongguk.edu (J.-S.S.)

* Correspondence: kadamavinash@dongguk.edu and avikadam2010@gmail.com (A.A.K.); Tel.: +82-31-961-5616; Fax: 82-31-961-5108

Methods S1: Characterizations

The morphology of the α-Cellulose-Fe_3O_4-CTNs-SH was observed by high-resolution transmission electron microscopy (HR-TEM) analysis (Tecnai G2 transmission electron microscope, Hillsboro, OR, USA). The sample for HR-TEM analysis was repapered by ultra-sonicating the α-Cellulose-Fe_3O_4-CTNs-SH solution for 1 hour (Sonics Vibra-Cell VC130 Ultrasonic Processor, Sonics & Materials, Inc., Newtown, CT, USA). The sample was drop coated on the carbon HR-TEM grid. The crystalline nature of the α-Cellulose-Fe_3O_4-CTNs-SH was evaluated by the X-ray powder diffractometer (XRD, Cu-Kα radiation (λ = 1.5418 Å), Ultima IV/Rigaku, Tokyo, Japan) analysis. The α-Cellulose-Fe_3O_4-CTNs-SH magnetic properties was carried out by vibrating sample magnetometer (VSM, Lakeshore, Model: 7407, LA, USA). The surface elemental profile of α-Cellulose-Fe_3O_4-CTNs-SH-Laccase (in powder form were analyzed by X-ray photoelectron spectroscopy (XPS, Theta Probe AR-XPS System, Thermo Fisher Scientific, Dartford, UK). The functional group profile of samples; α-Cellulose-Fe_3O_4-CTNs-SH and α-Cellulose-Fe_3O_4-CTNs-SH-Laccase were analyzed by Fourier transform infrared spectroscopy (FT-IR, Spectrum 100, PerkinElmer, Waltham, MA, USA).
